# Castration of Cryptorchids (Ridglings)1Read before the annual meeting of the Pennsylvania State Veterinary Medical Association, March, 1899.

**Published:** 1899-05

**Authors:** J. F. Butterfield

**Affiliations:** South Montrose, Pa.


### CASTRATION OF CRYPTORCHIDS (RIDGLINGS),[1]

By J. F. Butterfield, V.S.,
SOUTH MONTROSE, PA.

The cryptorchid is a malformation in which the testicle (one or both) does not descend into the scrotum. It will be found inside the abdomen, detained in the inguinal canal, or descended to the flank or some obscure situation. The percentage of animals with this irregularity is quite small; probably not more than one-tenth of 1 per cent. My experience has been: I have found it most prevalent in the horse, next in the pig, and quite rare in bovines, having seen but two, and they were flankers. It is quite important that ridgling horses be castrated, because they are more liable to become vicious than complete horses.

[1] Read before the annual meeting of the Pennsylvania State Veterinary Medical Association, March, 1899.

It is not well to breed from them, owing to the hereditary tendency. In Susquehanna County a horse of that character has been used for stock purposes for some years. Quite a percentage of his colts were cryptorchids.

It is only in comparatively recent years that ridgling castration has been attempted with any degree of success. It is an operation which requires an intimate knowledge of the parts, both theoretical and practical, especially practical. The operator should know from the sense of touch the parts he comes in contact with, which only comes from practice. He should be able to follow the natural course of the descent of the testicle in making the abdominal openings in the horse; to detect the peritoneum when he comes in contact with it; to distinguish the testicle from a loop of the intestine; to separate the vas deferens from the ureters, and not make the sad mistake of removing a kidney for a testicle. He should be a natural mechanic; he should have a cool head and a steady nerve, and be able to invent the best possible plan with the means at hand to overcome the difficulties he may meet, for he will not find it all clear sailing.

I have never used anæsthetics in this operation, but would recommend an anodyne—a hypodermic injection of morphia, three to five grains, one-half hour before operating. No doubt the anæsthetic would be the more up-to-date idea, but it is difficult to always have an assistant that is skilled in its administration, and then we would have the added danger of anæsthesia.

Prepare the animal for the operation by dieting from twelve to twenty-four hours previous, allowing only bran-mashes.

To secure the horse in the recumbent position for the removal of the apparently absent member we use the Conkey harness, preferring a grass-plot on a little incline. Lay him with his head down the hill, with side uppermost you wish to operate. This causes the abdominal viscera to gravitate out of the way, lessening the pressure upon your opening.

Having previously examined the parts in a standing position, now look for the cicatrix if the history of case says one testicle has been removed, manipulate again for flanker, or inguinal detention, which can most usually be detected now. If a flanker, remove where you find it. If an inguinal canal, make an incision in the same place in the scrotum, as though it were a normal castration, having previously disinfected the skin and hands with the usual bichloride or lysol solutions, and instruments with carbolic or formaldehyde solution. Make the incision large enough to introduce the hand. An operator with a small hand has an advantage. Now tear and stretch the fascia outward from the incision, and with a rotary motion with the hand partially closed, put aside the muscles next to the skin in the direction of the descent of the testicle to the inguinal ring, where you will find the testicle detained in the ring. Bring it to the surface and remove it with the écraseur in the usual manner.

Now, for the abdominal ridgling you make the same incision and same entrance to the ring as in the detained ring operation. You now pass above the ring two or three inches and break the peritoneum with one finger. Introduce two fingers, and usually you will detect the vas deferens right where you break through. Follow out to the testicle and bring it to the surface. Invariably the testicle is small and flabby. Remove with the écraseur. Should you not be able to locate the vas deferens as described, then enlarge the opening into the abdomen sufficient to introduce the hand. Pass above the bladder to the vesiculæ seminales, follow the vas deferens to the testicle, and bring outside. Until you become accustomed to this operation you will find it a help to introduce one hand into the rectum. It will aid in guiding you to the parts you wish to find. Should it be a double ridgling, do not open both sides, but pass your hand across from the first opening and locate the vas deferens as before, and bring to the surface, or nearly so, as the cord may be a little short. You can bring it up sufficient to pass the chain over it.

Some may ask, Will not the testicle descend when detained in the inguinal ring? In some cases it does. It is a question, however, whether it will pay the owner the necessary expense and time to wait.

In this operation upon the pig, which we are frequently called upon to perform, I suspend him by the hind legs, open the abdominal cavity about one inch to the right of the penis, making the incision two or three inches long. This will bring into view the bladder and vas deferens, which you trace to the testicle. Remove in the usual manner, and close the opening with three or four stitches, using a full curved needle, engaging skin, muscle, and peritoneum in each stitch.

*After-treatment.* Do not let the horse lie down and roll for eighteen hours after the operation. Feed hay sparingly for two days. Should there be much swelling, foment with clean warm water, in which use some germicide. Give walking exercise every day after the first day. Should a high temperature present itself treat with the usual fever remedies.

In the pig remove the stitches in ten days.

